# Exploring unobserved household living conditions in multilevel choice modeling: An application to contraceptive adoption by Indian women

**DOI:** 10.1371/journal.pone.0191784

**Published:** 2018-01-31

**Authors:** José G. Dias, Isabel Tiago de Oliveira

**Affiliations:** 1 Instituto Universitário de Lisboa (ISCTE-IUL), BRU-IUL, Lisboa, Portugal; 2 Instituto Universitário de Lisboa (ISCTE-IUL), CIES-IUL, Lisboa, Portugal; University of Exeter, UNITED KINGDOM

## Abstract

This research analyzes the effect of the poverty-wealth dimension on contraceptive adoption by Indian women when no direct measures of income/expenditures are available to use as covariates. The index–Household Living Conditions (HLC)–is based on household assets and dwelling characteristics and is computed by an item response model simultaneously with the choice model in a new single-step approach. That is, the HLC indicator is treated as a latent covariate measured by a set of items, it depends on a set of concomitant variables, and explains contraceptive choices in a probit regression. Additionally, the model accounts for complex survey design and sample weights in a multilevel framework. Regarding our case study on contraceptive adoption by Indian women, results show that women with better household living conditions tend to adopt contraception more often than their counterparts. This effect is significant after controlling other factors such as education, caste, and religion. The external validation of the indicator shows that it can also be used at aggregate levels of analysis (e.g., county or state) whenever no other indicators of household living conditions are available.

## Introduction

The modeling and understanding of social and health phenomena are heavily dependent on socioeconomic measures, i.e., the economic resources available to individuals and households. In most theoretical frameworks, the socioeconomic dimension needs to be controlled as a covariate and methods are therefore required to estimate the economic resources available to individuals and households. These resources can be divided into material wealth and intangible resources such as education and skills [1]. Income and consumption data are the most popular measures of material wealth or standards of living [2]. Income refers to the earnings from productive activities and current transfers; consumption refers to resources actually consumed and is expressed by expenditure data. Measured income often diverges from measured consumption as it is possible to save from income and to finance consumption from borrowing [3]. Despite a lively debate on which is the best measure of material wealth, there is some agreement that the smooth nature of consumption makes it the most suitable measurement of the economic component of living standards [2,4]. Moreover, less developed countries often report inaccurate income data, which are further masked by various forms of informal earning mechanisms, such as self-employment and economic activities within and outside the household, particularly in rural settings. In these contexts, it is generally far easier to measure consumption than income [2].

Many surveys in developing countries do not collect data on income or expenditures as they tend to be unreliable and lack standards for comparison between socioeconomic groups. Nevertheless, the Demographic and Health Surveys (DHS) collect and disseminate accurate and standardized data on household assets and dwelling characteristics for nationally representative samples. In addition to these indicators, these surveys collect data on fertility, reproductive health, maternal and child health from about 90 countries. The sampling design and survey instruments are standard across countries, thus allowing for cross-country analyses. Additionally, the DHS data sets are accessible to users including academic researchers and those from national and international agencies, at http://dhsprogram.com/. As a result, the DHS has become a standard source for international research on demography and health (particularly maternal and child health) in developing countries. Notwithstanding, although expenditure data is very useful, it is not widely collected in retrospective surveys. DHS is no exception to this.

These international surveys collect systematic data on household assets and dwelling characteristics (e.g., radio, TV, car, access to drinking water, type of toilet facility, roof material). Although it is important to acknowledge the range of variables available for the measurement of household wealth, it is often difficult to encapsulate all variables into a single score variable and also measure each one with respect to the outcome variable. Some researchers have proposed measures of material wealth [5–8]. While the variables considered in the construction of a wealth index are not based on any valid theoretical foundations, they provide a proxy to represent the socioeconomic dimension. It has been shown that this type of indicator is a reliable measure of expenditures [9–11]. For instance, Tasciotti and Wagner [12] compare census and survey data for Malawi and conclude that “the LSMS and DHS data are not only highly comparable but also representative as demonstrated by the comparison with the 2008 census” (p. 23). Recently, Batana [13] took the broader perspective of Sen’s definition of poverty rather than using the poverty-wealth dimension. He goes beyond defining poverty on the basis of material assets by adding other indicators such as schooling, BMI (body mass index), and empowerment.

The literature offers at least two approaches in which a range of household assets and related dwelling characteristics are weighted in the overall index: a) the *a priori* approach in which the index results from a sum of indicator or dummy variables for whether a household possesses certain assets [5,9]; b) the *a posterior* approach that deals with latent variables or underlying dimensions and weights are factor loadings.

Different techniques have been applied to DHS data sets. The most common techniques used to derive *a posterior* scores are: principal component analysis [8,14] and factor analysis [6,15]. Booysen et al. [16] argue that multiple correspondence analysis is more appropriate for the non-metric nature of observed data.

The DHS data set provides the Wealth Index (WI), originally introduced by Filmer and Pritchett [8]. This index measures the poverty-wealth dimension at the household level using the first dimension/factor results from a principal component analysis (based on the household assets and dwelling characteristics). An important feature of the DHS data is that the individual-level demographic information can be easily linked to household socioeconomic data collected at the time of the survey. Additionally, DHS surveys make the WI available as an indicator of households’ socioeconomic dimension [17]. It has become usual to use this index to address the poverty-wealth dimension in demographic and health research in developing countries, because the WI is included in the Demographic and Health Surveys databases available for scientific research.

Conceptually speaking, it is particularly interesting to assume that although household living conditions cannot be observed directly, the level of this latent variable is reflected in a set of manifest or observed variables [18]. Factor analysis is just one of these latent variable models but it is inappropriate for modeling household living conditions because it assumes that both latent and manifest variables are continuous. Indeed, most variables in the Demographic and Health Surveys that measure dimensions of household living conditions are collected using nominal and ordinal scales of measurement, and hence nonmetric (discrete) data. We conceptualize Household Living Conditions (HLC) as a continuous latent variable that is measured by an item response theory (IRT) model [18–19]. IRT focuses on the development of an accurate battery of items to measure and score tests. It was first proposed in the field of psychometrics for the purpose of ability assessment. It is used in social sciences (namely education and psychology) to measure different kinds of ability (e.g., foreigner language skills) or more general traits (e.g., intelligence, consumer behavior, attitudes). The manifest variables are nonmetric (e.g., binary, ordinal), which makes it a popular alternative to factor analysis and principal component analysis in health and social sciences, for example [20–21]. The IRT methodology is also fundamental in the assessment of international programs [22]. For example, in the context of poverty measurement, IRT was applied in Spain and Malawi to measure household wealth [23–24].

This research combines latent variable modeling with choice modeling, taking Household Living Conditions (HLC) as a latent covariate. Thus, our proposal integrates both analyses into a single-step model using a probabilistic framework. Contrary to Oliveira and Dias [25] and Oliveira et al. [26] in which the WI provided by the DHS database was used to capture the poverty wealth impact on contraception adoption and to discriminate different contraceptive methods, respectively, this paper estimates the household living conditions and the choice model, simultaneously. Additionally, the latent variable HLC can be explained by covariates.

Our application employs this new method to the study of the impact of household living conditions on the most important long-term variable in population dynamics: fertility. The study of fertility in India is crucial to the whole World. The United Nations Population Prospects [27] estimate that India will soon become the most populated country in the World, surpassing China, as a result of both a very young population structure and fertility above the replacement level. Despite successive government efforts to promote family planning since the second half of the 20th century [28,29], India continues to have a comparatively high level of fertility even by Asian standards. In fact, fertility in India is currently above the average for Asia and, notably, for China (2.44 children per woman in 2010–15 vs. 2.20 and 1.60 respectively [27]).

This research aims to integrate a non-demographic complex and multidimensional factor (the poverty-wealth dimension) with a demographic health outcome (fertility regulation by means of contraception). The association of wealth and health is relevant in social sciences and epidemiology. The specific relation between contraception and the socioeconomic dimension is the subject of numerous studies in developing countries, frequently within the context of maternal health research [30–32]. Studies on contraceptive behavior and the socio-economic situation in developing countries reveal important differentials associated with the wealth dimension, education, and other socio-economic characteristics. Overall, multivariate analyses that simultaneously include women’s education (a usual proxy for SES–Socioeconomic status) and wealth (measured by the classic Wealth Index) demonstrate that both affect contraceptive adoption, even after controlling for other factors. The better off tend to adopt contraception more frequently than their counterparts [30–33].

The paper is structured as follows. The next section describes the methodology for estimating the impact of a latent covariate on the dependent variable, controlling other observed covariates. A case study then addresses the impact of the socioeconomic context on the choice of contraceptive methods by Indian women. The purpose of this analysis is to take a latent variable approach based on household characteristics to estimate the impact of household living conditions on contraception adoption. Results are validated by comparing our estimates with official statistics from India. The paper concludes with further potential extensions and applications of this integrated framework.

## Multilevel choice modeling with a latent covariate

**Fig 1:**
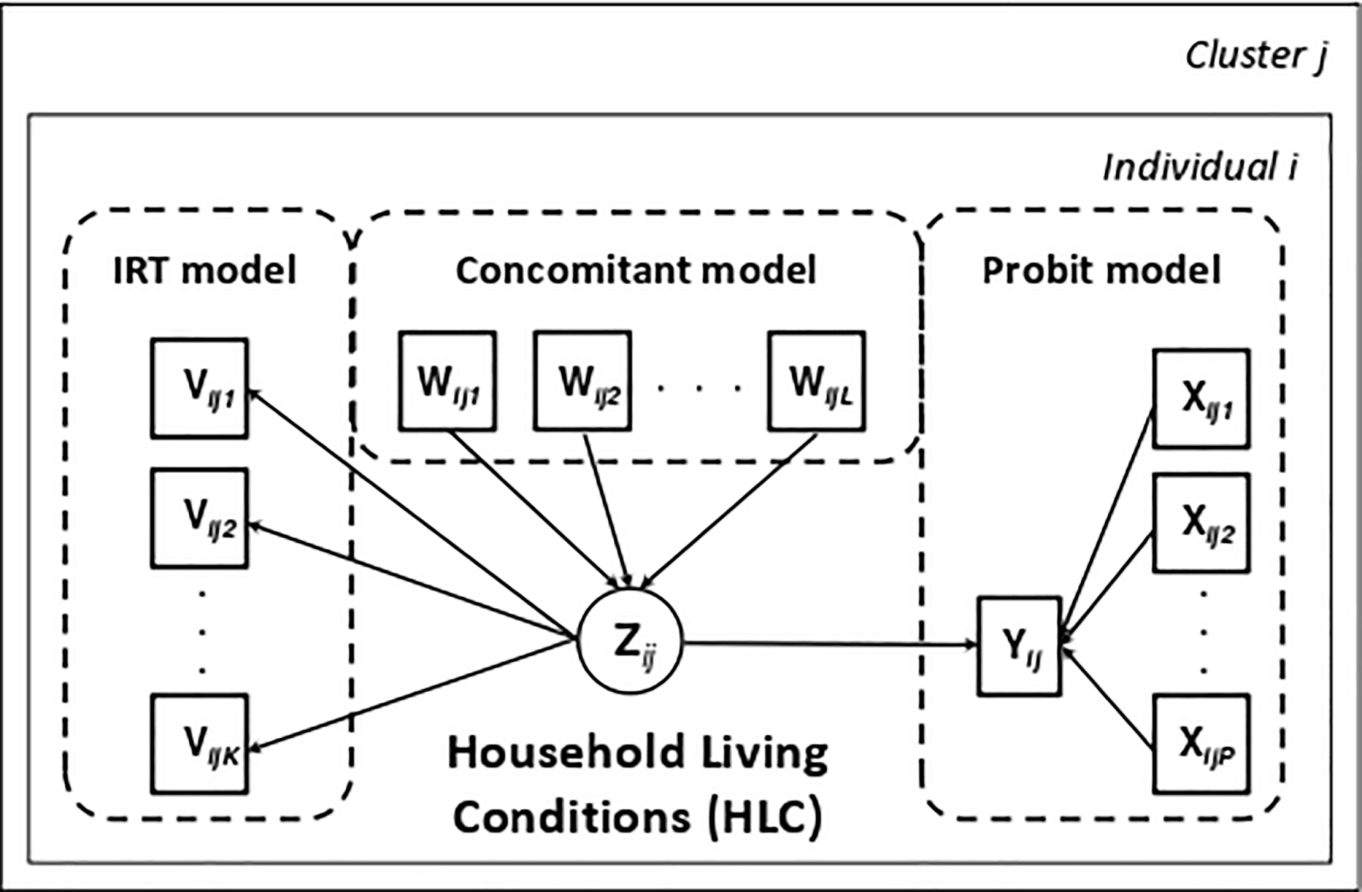
The multilevel choice model with a latent covariate.

The proposed framework takes the form of a probit regression model with a latent covariate, more specifically, the Household Living Conditions (HLC) indicator, measured by a set of items using an Item Response Theory (IRT) model. Most surveys tend to collect data at different levels of the hierarchy using complex sampling. For example, individuals may be clustered within regions or countries. In this context, the traditional assumption of independence is violated and this nesting structure needs to be addressed using multilevel modeling [34–36]. The proposed multilevel probit regression model with a latent covariate is depicted in Fig 1 for observation i in cluster *j*, where boxes show observed variables and the circle represents the latent variable. The total number of units of the upper level is indicated by N and within cluster *j* is designated by *n*_*j*_. The total sample size is $=\sum_{j=1}^{N}n_{j}$.

The binary dependent variable is Y_ij_ and is explained by the latent variable *z*_*ij*_ and a set of *P* observed covariates (***x***_*ijp*_). Let *p*_*ij*_ be the probability of success for observation *i* in cluster *j*, i.e., *p*_*ij*_ = *P*(*Y*_*ij*_ = 1 | ***x***_*ij*_, *z*_*ij*_). This binary model defines a latent variable $Y_{ij}^{*}$ and a threshold value of *τ*: we observe a success if $Y_{ij}^{*}>\tau$, i.e., in this case *Y*_*ij*_ = 1. The linear component of the model is given by $Y_{ij}^{*}=x_{ij}^{\prime }\beta +\gamma z_{ij}+u_{j}+\epsilon_{ij}$, where x_ij_ is the vector that contains the *P* observed covariates for observation *i* in cluster *j*, ***β*** is the vector of regression parameters (fixed effects), *γ* is the parameter of the linear effect associated to the latent household living conditions indicator (loading), *z*_*ij*_ is the latent household living conditions, *u*_*j*_ is the random effect for cluster *j*, and *ϵ*_*ij*_ is the error term. The threshold replaces the intercept in the model, whereas the random effect (*u*_*j*_) represents factors affecting $Y_{ij}^{*}$ that are shared by all units within cluster *j* after controlling individual covariates and the latent factor. The probit regression framework assumes standard normal errors and random intercepts (*u*_*j*_) are independent of the errors *ϵ*_*ij*_ and normally distributed: $u_{j}∼N(0,\sigma_{u}^{2})$.

This single-step approach is completed with the definition of the latent variable, household living conditions (HLC), measured by a set of *K* observed items (*v*_*ijk*_, *k* = 1,…, *K*). This model can be interpreted as a factorial model with a continuous latent variable and discrete manifest variables (see Fig 1) and when used autonomously, it is called the IRT model [19,37]. The IRT specification here uses the factor-analytic parameterization, which is similar to the $Y_{ij}^{*}$ specification, i.e., it is given by the loading and threshold parameters for each item. The traditional 2-P definition of the IRT can be derived from this factor analytic specification [38]. The difficulty and discrimination of the item are given by the ratio threshold/loading and loading, respectively. The difficulty parameter in the present context indicates how rare the item is in the household. The discrimination parameter is a measure of an item’s differential capability, i.e., a high discrimination parameter value suggests an item that has a strong ability to differentiate households. For each binary manifest variable *k*, we estimate the threshold and the loading parameters. Like in factor analysis, we assume that this latent variable score follows a normal distribution. The variance of the latent variable is fixed at 1 to maintain the coefficients of the latent variable identified (*γ*). Because the score may vary for different contextual variables, this model allows distinct control variables *w*_*ijl*_, where *l* = 1,…, *L*. Thus, *z*_*ij*_ has expected value *θ*_1_*w*_*ij*1_ + ⋯ + *θ*_*L*_*w*_*ijL*_ and unit variance, where *θ*_*l*_ measures the impact (slope) of concomitant variables *w*_*l*_ on the *z*_*ij*_ (HLC). Note that the intercept is zero so that the model remains identified and the slopes provide the departure from the reference category. This submodel is called the concomitant regression model.

The model was estimated using the maximum likelihood method using MPlus. This computes maximum likelihood estimates with standard errors given by the sandwich estimator that is robust to non-normality and non-independence of observations [39, p. 533]. The complex design of the sample (weights) was taken into account [40].

## A case study: Modeling contraceptive adoption in India

### Population, sample, and variables

We apply the integrated model to data from the Indian National Family Health Survey (NFHS) from 2005–06 (NFHS-3) [41]. The NFHS provides a representative nationwide sample of Indian women. This data set was downloaded from the official website of the DHS program (https://dhsprogram.com), after obtaining permission from the DHS team. The Demographic and Health Surveys (DHSs) are free and public data sets. Researchers have to register with MEASURE DHS and submit the request before access to DHS data is granted. This is the most recent survey with a representative sample on the Indian population providing data for research purposes (a new DHS is now ongoing in India, but no data are available yet). This survey belongs to the DHS series and covers a large number of questions on women’s fertility and contraceptive practices, maternal and infant health, in addition to the usual individual sociodemographic characteristics and the household assets and dwelling characteristics.

The original database with all women of fertile age was reduced to a smaller one with 31197 cases. The aim was to focus only on the women that may, or not, need to use family planning methods. In many Asian countries, including India, contraception is largely an issue for married women as unmarried women are not expected to engage in sexual relations [42]. For instance, 99.3% of the women in the sample who answered questions on contraception were married and only 0.7% unmarried women had sexual experience (own computation based on values presented in [41, p. 121]). We select only fecund married women (with non-sterilized husbands) with sexual experience and living in the household (excluding the “not the de jure population”). This new data set only includes unsterilized and recently sterilized women as the association between the current socioeconomic situation and contraceptive behavior cannot be established if sterilization took place a long time ago. On the other hand, the issue of endogeneity must not be overlooked; in addition to the influence of household living conditions on women’s contraceptive adoption, contraceptive choices can also have reciprocal effects. This selection of a subsample minimizes these effects.

The dependent variable, current use of contraception, is denoted by Y_ij_ and is coded as either 1 (success: use of contraception) or 0 (failure: no use of contraception). Thus, it is binary: the contraceptive users may have adopted any traditional or modern method and non-users used no form of family planning at the time of the survey. When examining the marginal impact of the household living conditions on women’s contraceptive adoption, we need to control for the effects of other variables in the model. We examine the effects of life cycle variables (age, number and sex composition of offspring), residence (urban vs. rural and nuclear vs. joint households), and other socioeconomic and cultural factors (caste system, religion, education and occupation). Thus, apart from material wealth, we control for other types of wealth (e.g., social capital) that may have an impact on contraceptive adoption. For instance, both education and wealth index tend to be included as covariates in the context of India (see, e.g., [43,44]), and even in analyses with a broader geographical spectrum (see [33]).

A set of items is used to measure the latent variable. The items include dwelling characteristics i.e. type of flooring, type of toilet facility, cooking fuel, household electrification, glass windows, as well as household assets such as a pressure cooker, telephone, color television, refrigerator, computer, car, and motorcycle/scooter. The binary variable, urban/rural, was added to the model as a concomitant variable. It has been shown that there is a difference in the distribution of the Wealth Index in rural and urban environments in many countries, and India is no exception (e.g., [45]).

The community or place of residence (Primary Sampling Unit (PSU)) constitutes the upper level in this multilevel model taking into account the hierarchical structure of data and adjusting for the community effects. Sample weights at the household level are included in the NFHS data and are based on the complex sample design of the survey.

### Descriptive findings

The sample description (Table 1) shows that the large majority of women in the sample live in rural areas. Contraceptive prevalence is clearly lower in rural than urban settings. A relatively high proportion of women from nuclear families use contraception, but these women comprise less than half of the sample. Women in the middle of the fertile ages are the most typical users of contraception and family planning methods; this is closely linked with the number and sex composition of offspring. Religion is another important factor, and Muslim women use contraception less frequently. Contraceptive prevalence is also lower among women from scheduled castes, tribes, and other backward classes than for women that do not classify themselves in any of these categories. For female education, there is a strong gradient for the adoption of family planning methods: contraceptive prevalence rises as the level of women’s education increases.

**Table 1:** Socio-economic factors and current contraceptive method.

		Count^a^	%^b^	Contraceptive Prevalence %^b^
Caste	Scheduled caste	5321	19.5	57.6
	Scheduled tribe	4292	8.3	47.8
	Other backward class [OBC]	10217	40.2	55.5
	None of them	11367	32.1	63.4
Place of residence	Urban	13935	31.8	65.9
	Rural	17262	68.2	54.1
Religion	Hindu	23250	81.7	58.7
	Muslim	3887	13.3	51.7
	Other	4060	5.0	59.8
Household structure	Nuclear	14551	45.0	63.7
	Non-nuclear	16646	55.0	53.0
Female age	Less than 25 yr	10147	37.1	40.0
	25–34 yr	16236	49.2	69.8
	35 yr or more	4814	13.7	63.0
Female education	No formal schooling	10223	41.4	52.0
	Primary	4309	14.1	57.1
	Secondary	13067	36.4	62.0
	Higher	3598	8.1	70.0
Female occupation	Working	11450	38.6	57.6
	Not-Working	19747	61.4	58.0
Living children	0	3933	13.4	12.1
	1	7013	20.8	46.2
	2	9222	28.0	73.0
	3	5126	16.6	72.6
	4+	5903	21.1	66.5
Living boys	0	9597	30.1	33.1
	1	12047	37.3	64.7
	2	6590	21.8	76.9
	3	1957	7.0	68.5
	4+	1006	3.8	57.6
**Total**		**31197**	**100.0**	**57.8**

^a^Number of respondents are based on unweighted data.

^b^Percentages are sample weight-adjusted.

### Measurement of Household Living Conditions (HLC)

Table 2 shows the estimates of the IRT component, accounting for the measurement of the latent variable: HLC. All indicators are significant in the measurement of the latent variable. The HLC has a marked impact on the possession of goods like color television (1.566), refrigerator (1.514), pressure cooker (1.476), cooking with good fuel (1.734), and household electrification (1.457). These items are best able to discriminate between households. The same positive effect, though not so strong, is reflected in the following items: flush toilet (1.309), finished flooring (1.125), glass windows (0.995), and owning a computer (1.156), a telephone (1.100), or a car (1.015). Regarding the difficulty parameter, which indicates the rarity of a characteristic or asset, we conclude the items are scaled from the most common namely household electrification, i.e., most of the households have access to it (aggregate column: 77.6%) to the most scarce i.e. ownership of a car (3.161) and ownership of a computer (3.228) that are available in 5.5% and 4.3% of the households, respectively. To sum up, this factorial model presents the unidimensional structure of the HLC latent variable.

**Table 2:** Item response model.

Variables	Aggregate	Loadings			Thresholds			Difficulty	Discrimination
		Estimate	S.E.	p-value	Estimate	S.E.	p-value		
Household electrification (Yes)	0.776	1.457	0.037	0.000	-0.658	0.038	0.000	-0.452	1.457
House has windows with glass (Yes)	0.244	0.995	0.022	0.000	1.744	0.037	0.000	1.753	0.995
Type of toilet facility (Flush toilet)	0.518	1.309	0.027	0.000	0.747	0.038	0.000	0.571	1.309
Type of flooring (Finished)	0.532	1.125	0.024	0.000	0.581	0.030	0.000	0.516	1.125
Cooking fuel (Good)	0.352	1.734	0.037	0.000	2.042	0.057	0.000	1.178	1.734
Ownership of a pressure cooker (Yes)	0.551	1.476	0.030	0.000	0.664	0.037	0.000	0.450	1.476
Ownership of a colour television (Yes)	0.373	1.566	0.031	0.000	1.721	0.040	0.000	1.099	1.566
Ownership of any telephone (Yes)	0.189	1.100	0.026	0.000	2.203	0.043	0.000	2.003	1.100
Ownership of a computer (Yes)	0.043	1.156	0.040	0.000	3.732	0.091	0.000	3.228	1.156
Ownership of a refrigerator (Yes)	0.240	1.514	0.036	0.000	2.477	0.056	0.000	1.636	1.514
Ownership a car (Yes)	0.055	1.015	0.036	0.000	3.208	0.072	0.000	3.161	1.015
Ownership a motorcycle/scooter (Yes)	0.247	0.864	0.020	0.000	1.559	0.027	0.000	1.804	0.864

The concomitant component of HLC is given in Table 3. As the place of residence plays an important role, we allow that the distribution of the HLC is different for women living in rural and urban areas. We conclude that the HLC score for urban households is on average 1.437 higher than for rural households, and the difference is statistically significant.

**Table 3:** Regression models (choice and concomitant models).

Variables	Estimate		S.E.	p-value
**Level 1—Regression model: Fixed effects**^a^				
Household Living Conditions (HLC)				
Linear	0.202		0.017	0.000
Quadratic	-0.006		0.007	0.381
Female age				
Linear	0.143		0.012	0.000
Quadratic	-0.003		0.001	0.000
Caste (ref: None of them)				
Scheduled caste	-0.037		0.029	0.195
Scheduled tribe	-0.318		0.038	0.000
Other backward class	-0.086		0.024	0.000
Residence (ref: Rural)				
Urban	0.077		0.028	0.007
Religion (ref: Hindu)				
Muslim	-0.324		0.034	0.000
Other	-0.270		0.037	0.000
Houshold structure (ref: Non-nuclear)				
Nuclear	0.086		0.019	0.000
Female education (ref: No formal schooling)				
Primary	0.157		0.028	0.000
Secondary	0.324		0.026	0.000
Higher	0.519		0.042	0.000
Female occupation (ref: Not working)				
Working	0.084		0.02	0.000
Living children (reference: 0)				
1	1.017		0.038	0.000
2	1.606		0.043	0.000
3	1.721		0.049	0.000
4+	1.782		0.054	0.000
Living boys (reference: 0)				
1	0.263		0.025	0.000
2	0.517		0.033	0.000
3	0.448		0.044	0.000
4+	0.329		0.056	0.000
Thresholds	3.476		0.176	0.000
**Level 1—Concomitant model**^b^				
Slope (Urban)	1.437		0.034	0.000
**Level 2—Random effects**				
*Var*(*u*_*j*_)	0.230		0.014	0.000
Log-likelihood value	-152534.760			

^a^Residual variance equals 1.

^b^Intercept and variance of HLC are 0 and 1, respectively.

### Contraceptive choice results

The tendencies observed in this first description are analyzed by means of a multilevel probit regression model with a latent covariate. This probit model estimates the impact of HLC and controls for other factors (e.g., life cycle variables and residence factors). We note that the impact of age and HLC on $Y_{ij}^{*}$ is specified to be quadratic. Thus, for instance, for HLC (*z*_*ij*_) we have $\gamma_{linear}z_{ij}+\gamma_{quadratic}z_{ij}^{2}$ in the linear component of the model. These joint effects of household living conditions on the regression model are particularly important. If we fail to reject *H*_0_: *γ*_*linear*_ = *γ*_*quadratic*_ = 0, HLC, which is measured by a set of indicators and explained by the concomitant variable urban, cannot explain the dependent variable. A second model under the null hypothesis was estimated. Based on the likelihood ratio statistic that follows the qui-square distribution, the p-value is <10^−6^. And the decision is to reject the null hypothesis. Thus, HLC has a joint effect on the contraceptive adoption.

Results from the multilevel probit model for contraceptive adoption in India reveal the impact of HLC plus a set of covariates on contraceptive use (Table 3). More specifically, the latent variable HLC has a significant and linear impact on contraceptive adoption: as HLC increases, the probability of adopting contraception also increases. The non-linear impact is not significant.

Previous research on the contraceptive behavior of Indian women reveals that contraceptive use is quite sensitive to the number and sex composition of previous births [46–48] and that Muslim women adopt contraception less frequently [49,50] as do those from disadvantaged social groups [51,52], those living in non-nuclear households [53], and those living in rural areas [41]. On the other hand, socioeconomic factors, e.g. wealth [25,54] and women’s education [43,55,56], proved important to the adoption of family planning.

Our results show that age has a non-linear effect on contraceptive adoption: there is almost an inverted U shape relation, with the greatest likelihood of adopting contraception coming in the most fecund ages. The number and sex composition of offspring are important factors for the adoption of family planning methods. The residence is also a significant factor: the probability of women living in urban areas and in nuclear households using contraception is higher than that of their counterparts. Turning to India’s traditional socioeconomic and cultural differences, it is clear that women from scheduled tribes and other backward classes were less likely to adopt family planning than women in the reference category. On the other hand, both Muslim women and women from other religious affiliations have a lower probability of using contraception than Hindu women. Additionally, female work and education both increase the odds of adopting family planning methods. As expected, the education gradient is very clear. To sum up, Hindu women and women not belonging to marginal communities are the most likely to control their fertility. On the other hand, women living in nuclear households are more likely to use contraception than their counterparts as are women living in urban settings. Nevertheless, it should be noted that women living in rural settings constitute the biggest group in the Indian population.

The intraclass correlation (ICC) corresponds to the proportion of the total variability that is explained by cluster level: $ICC=\sigma_{u}^{2}/(1+\sigma_{u}^{2})$. The upper level (PSU) explains 18.8% of the total variance.

**Fig 2:**
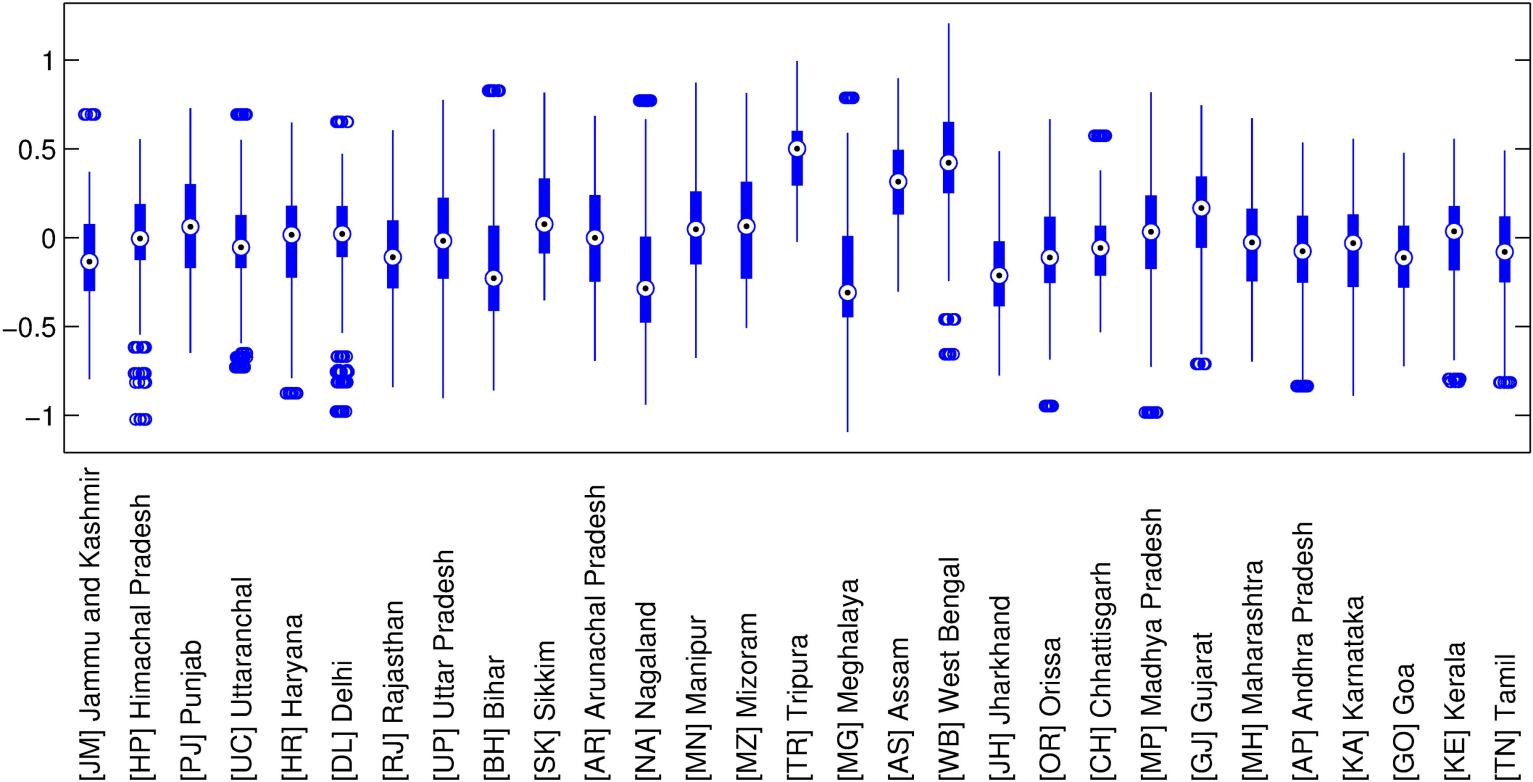
Distribution of the estimated random effects grouped by state.

Fig 2 depicts the boxplot of the PSU effects grouped by state. We notice that random effects control the spatial dependency in the multilevel structure. Its impact on the linear component of the model either adds or subtracts a common factor to all observations from the same PSU and corrects the impact of the fixed effects. States from Northeast India tend to have high absolute medians of the estimated random effect (e.g., Tripuna, Meghalaya, Assam, Nagaland). The same happens with states from Eastern India such as Jharkhand and West Bengal. These results show that these regions of India have specific characteristics (e.g., houses built with different materials) that are corrected by the random effect in this two-level structure.

**Fig 3:**
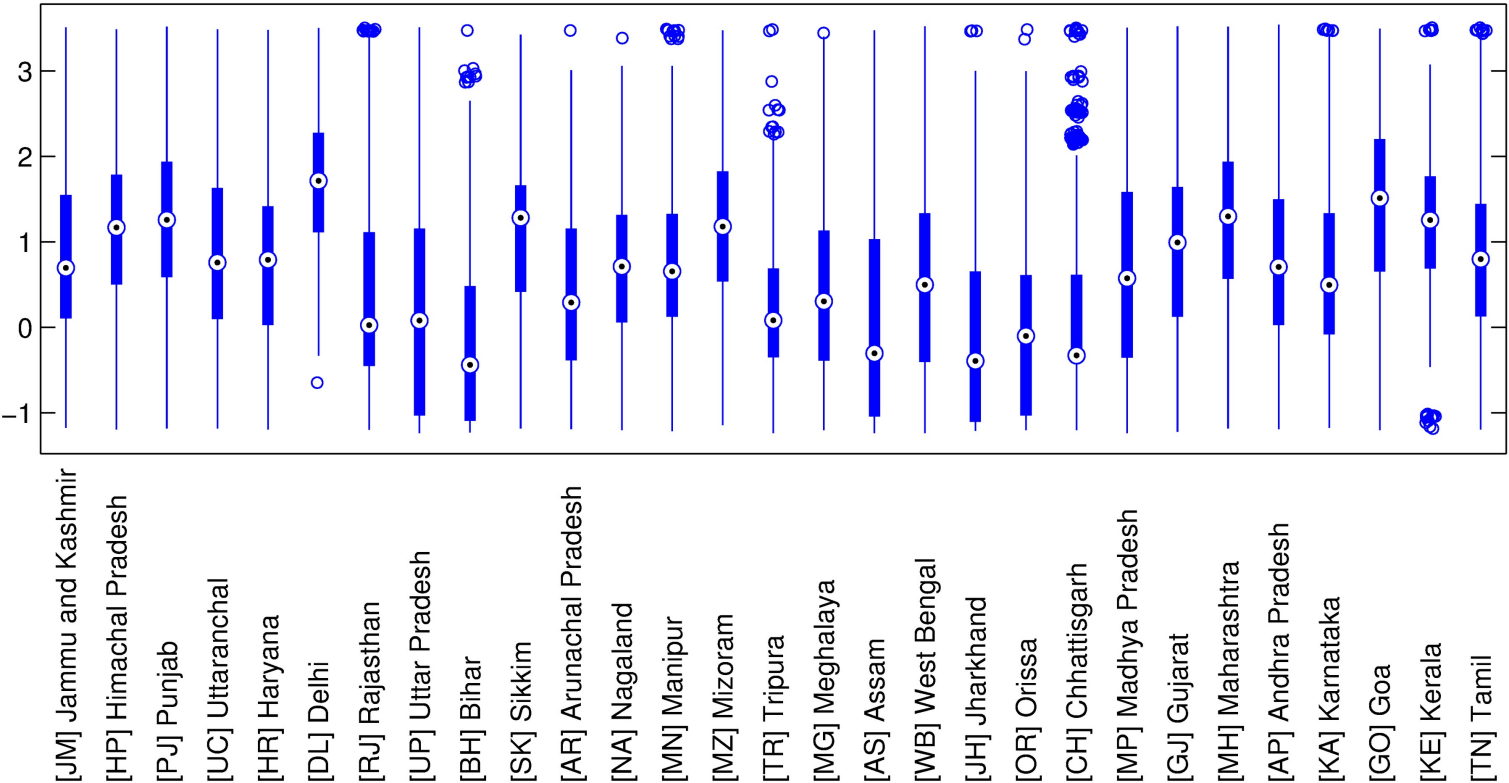
Distribution of Household Living Conditions (HLC) by Indian state.

Finally, Fig 3 shows the distribution of the HLC in each Indian state. We observe within- and between-state heterogeneity in terms of median and interquartile range, respectively. Some states, such as Bihar, Assam, Jharkhand, Orissa, and Chhattisgarh, have particularly poor HLC at the household level (median level), while others e.g. Delhi, Goa, Kerala, Sikkim, and Maharashtra, have a better median HLC than most Indian states. In Central and East states, scores of HLC are particularly heterogeneous (Uttar Pradesh, Assam, West Bengal, Jharkhand, Orissa, Madhya Pradesh, Rajasthan and Bihar), whereas HLC in the North and Northeast states (Delhi, Tripura, Manipur, Nagaland, Sikkim, Punjab, Himachal Pradesh) and Kerala and Chhattisgarh (in the South and in the East) are the most homogeneous. In short, the Central and Eastern states tend to be poor and more heterogeneous than the West states and some of the Northeast and North Indian states.

### External validation of the HLC

As an illustration and external validation, we compare the HLC score (aggregated at the state level) with the respective Net State Domestic Product (NSDP) per capita at constant prices (2004–2005) for each Indian state. External validation is important as it provides a measure of the predictive ability and generalizability of the indicator in a different (external) context [57]. Data on NSDP are provided by the Government of India [58]. Table 4 summarizes the mean scores and ranking for both indicators.

**Table 4:** Comparison of NSDPpc and living conditions for Indian states.

State	NSDPpc 05/06		Household Living Conditions (HLC)		
	Indian rupees	Ranking	Mean score	Ranking	Std. Deviation
[JM] Jammu and Kashmir	22.406	20	0.813	11	1.073
[HP] Himachal Pradesh	35.806	6	0.992	6	0.794
[PJ] Punjab	34.096	9	1.240	4	0.926
[UC] Uttaranchal	27.781	13	0.833	10	1.041
[HR] Haryana	40.627	4	0.804	12	0.966
[DL] Delhi	69.128	2	1.845	1	0.814
[RJ] Rajasthan	19.445	21	0.224	19	1.154
[UP] Uttar Pradesh	13.445	28	-0.044	25	1.082
[BH] Bihar	7.588	29	-0.484	29	0.846
[SK] Sikkim	29.008	12	0.925	8	0.916
[AR] Arunachal Pradesh	26.870	15	0.311	18	1.062
[NA] Nagaland	33.072	10	0.419	16	0.935
[MN] Manipur	19.341	22	0.646	15	0.900
[MZ] Mizoram	25.826	16	1.213	5	0.957
[TR] Tripura	25.688	17	0.113	21	0.885
[MG] Meghalaya	24.278	18	0.135	20	1.039
[AS] Assam	17.050	26	-0.126	26	1.101
[WB] West Bengal	23.808	19	0.055	23	1.057
[JH] Jharkhand	17.406	25	-0.311	28	1.048
[OR] Orissa	18.194	24	-0.155	27	0.938
[CH] Chhattisgarh	18.530	23	-0.007	24	0.973
[MP] Madhya Pradesh	15.927	27	0.095	22	1.054
[GJ] Gujarat	36.102	5	0.922	9	1.005
[MH] Maharashtra	40.671	3	0.932	7	1.070
[AP] Andhra Pradesh	27.179	14	0.348	17	0.861
[KA] Karnataka	29.295	11	0.667	14	0.972
[GO] Goa	80.844	1	1.456	2	1.049
[KE] Kerala	35.492	7	1.253	3	0.834
[TN] Tamil	34.126	8	0.735	13	0.918
India	26.015		0.293		1.132

Note: NSDPpc—Net State Domestic Product per capita (Indian rupees); Weighted mean score.

**Fig 4:**
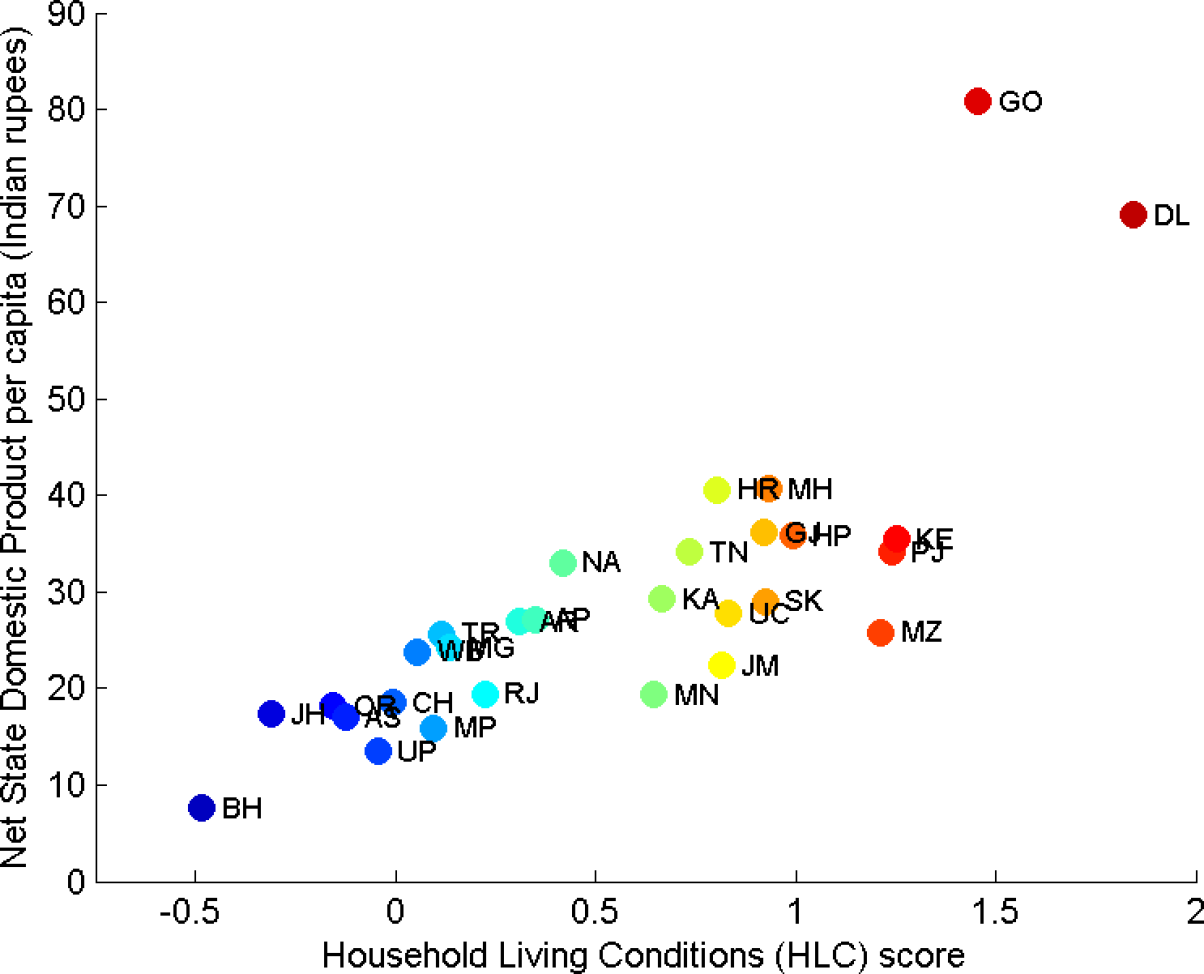
Relation between NSDPpc and Household Living Conditions (HLC) for Indian states (see Table 4 for the meaning of acronyms of Indian states).

Overall, we conclude that there is general agreement between the two indicators despite their conceptual difference. The HLC tends to be broader in scope than an income-based indicator. Fig 4 allows a more precise understanding of the relationship between these two variables. With the exception of the two small Indian states of Goa and New Delhi, which have the highest values, there is a strong linear relationship and Bihar occupies the bottom position. The Pearson correlation between the HLC and the NSDPpc of 0.794 indicates a strong association between these variables. In terms of rankings, the ordering of Indian states by the two indicators is also strongly associated (Spearman’s rho correlation = 0.853).

## Conclusion

This paper proposes an integrated choice modeling framework which adds covariates that are not measured directly. This is particularly important as most studies need to include control variables, e.g. the socioeconomic dimension of the phenomenon being explained. The model is embedded in a multilevel setting that takes the complex survey design into account.

The case study illustrates the approach by simultaneously estimating Household Living Conditions (HLC) as a latent covariate that explains a choice process in a probit regression. It addresses the association between contraceptive adoption and the women’s household position in terms of the poverty-wealth dimension in India. This relation is analyzed by allowing a latent covariate, the HLC indicator, into the model as an alternative to the standard wealth index (WI). The new indicator is estimated as part of the model simultaneously with the probit model for contraception. This research confirms that the household characteristics and assets are important predictors of women’s contraceptive behavior in India. Validation of the indicator by external data from a different source (Net State Domestic Product) shows that this new proxy is a valid measure of the material wealth. It also shows a promising application of the household-level scores to obtain an aggregate, for instance, at county- or state- level indicators that can be used to track poverty and inequality development goals where more specific data is lacking. This new single-step method to obtain indicators is more consistent at a methodological level than the usual WI and can be applied to other contexts, especially in empirical research using DHS or similar surveys. In particular this procedure overcomes the limitation of a lack of income/expenditure data to measure the socioeconomic dimension in surveys that collect household assets and dwelling characteristics (e.g., DHS and MICS (Multiple Indicator Cluster Surveys)).

From an empirical standpoint, the model can be used whenever the household living conditions construct is conceptualized as an unobserved covariate in social and health research. The fact that the model explicitly takes the socioeconomic dimension into account minimizes the problem of endogeneity between the dependent and the errors that may have biased the estimates in the model.

This model can be applied to contexts other than modeling the choice of contraception, e.g. to measure the impact of socioeconomic status on child undernutrition [59], HIV prevalence [60–62], women’s empowerment [63], and domestic violence [64]. Thus, this framework is a one-step alternative to the use of WI as an external covariate. Additionally, the definition of living conditions can be an extension of HLC by adding non-material items [13]. The IRT structure, measuring the HLC, could also be added to more complex contraception choice models [26].

This integrated choice modeling has several advantages, particularly in dealing with the endogeneity problems associated to the interrelated processes of wealth and health as it estimates LHC jointly. On the other hand, two limitations must be mentioned. First, this is a complex and sophisticated methodology and, consequently, is less accessible to a direct application by most researchers. Second, these indicators are specific to each application and embedded in the choice modeling with a specific dependent variable. Thus, this type of indicator should not be used in a different context, i.e., with another dependent variable; even with the same set of items, a new model should the estimated in a one-step approach.

Future research can also explore the application of this model to address highly correlated covariates. Aguilera et al. [65] proposed a logistic regression model with an embedded principal component structure for highly correlated covariates. It can be hypothesized that highly correlated covariates are manifestations of the same latent variable or construct. In this case, we can define an integrated factorial structure underlying the correlated covariates instead of using an external index construction based on the principal component analysis.
